# Obstructive sleep apnea and atrial fibrillation: insights from a bidirectional Mendelian randomization study

**DOI:** 10.1186/s12920-022-01180-5

**Published:** 2022-02-16

**Authors:** Lu Chen, Xingang Sun, Yuxian He, Yunlong Lu, Liangrong Zheng

**Affiliations:** Department of Cardiology and Atrial Fibrillation Center, The First Affiliated Hospital, School of Medicine, Zhejiang University, 79 Qingchun Road, Hangzhou, Zhejiang Province People’s Republic of China

**Keywords:** Obstructive sleep apnea, Atrial fibrillation, Mendelian randomization, Bidirectional, Causality

## Abstract

**Background:**

Observational studies have suggested that obstructive sleep apnea (OSA) is in relation to atrial fibrillation (AF); however, these studies might be confounded and whether the relationship is causal remains unclear. We conducted a bidirectional Mendelian randomization (MR) study to clarify the causal inference between OSA and AF.

**Methods:**

Genetic instruments for OSA and AF were obtained from genome-wide association studies. The fixed-effects inverse-variance weighted (IVW) method was used as the main method, supplemented by several sensitivity analyses. For replication, another AF dataset was used to validate the causal effect of OSA on AF. Furthermore, multivariable MR analyses were performed to obtain direct estimates adjusting for potential confounders.

**Results:**

Genetic liability to OSA was found to be significantly associated with a higher AF risk in the fixed-effects IVW method [odds ratio (OR) 1.210; 95% CI 1.119–1.307; *P* = 1.51 × 10^–6^]. The results were consistent in MR sensitivity analyses as well as in replication analyses, and the significance remained after adjusting for potential confounders. In the reverse MR analyses, there was no causal effect of AF on OSA.

**Conclusions:**

Our study strengthened the causal evidence of genetically predicted OSA with a higher AF risk. Early screening and appropriate management of OSA might show anti-arrhythmic benefits.

**Supplementary Information:**

The online version contains supplementary material available at 10.1186/s12920-022-01180-5.

## Background

Atrial fibrillation (AF), the most prevalent sustained cardiac arrhythmia worldwide, affects 2% to 4% of the adult population [1]. Due to an aging population and increased prevalence of risk factors, AF is associated with high morbidity and mortality, and increasingly contributes to a significant burden to patients, physicians, and healthcare systems globally [2].

Obstructive sleep apnea (OSA), characterized by the repetitive partial or complete collapse of the upper airway during sleep [3], is the most common type of sleep-disordered breathing and is highly prevalent. Previous studies suggested that OSA and AF were closely interconnected. The prevalence of OSA was higher in patients with AF compared with the general population [4]; conversely, the prevalence of AF in patients with sleep apnea was much higher than control participants [5]. Recent data showed that OSA is a potential risk factor of AF [6], and could exert a negative impact on the efficacy of catheter ablation and pharmacological antiarrhythmic therapy [7, 8]. In addition, OSA and AF share many risk factors and comorbidities, such as increasing age, male gender, obesity, hypertension, heart failure, and coronary artery disease [9, 10], making observational studies possibly biased. Considering the confounding factors and reverse causation inevitable in observational studies, the causality between OSA and AF remains unclear and warrants further investigation.

Mendelian randomization (MR) study, utilizing genetic variants as instrumental variables (IVs) to analyze the causal inference between exposures and outcomes, is less vulnerable to residual confounding [11]. Therefore, we conducted a two-sample bidirectional and multivariable MR study to estimate the causal inference between OSA and AF.

## Material and methods

### Study design

**Fig. 1:**
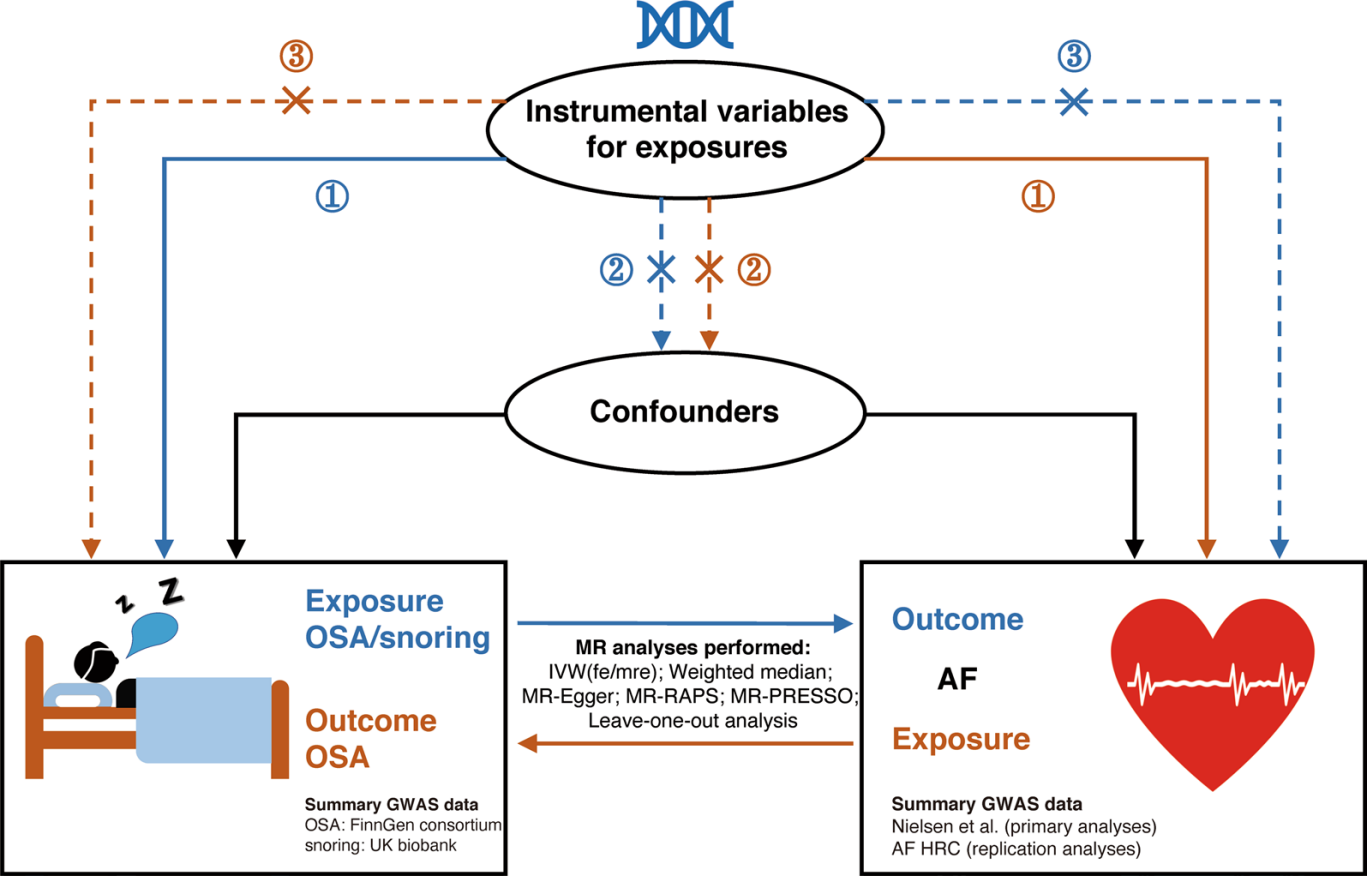
Schematic illustration of the bidirectional Mendelian randomization study on the causal inference between obstructive sleep apnea and atrial fibrillation. *Notes*: Mendelian randomization approach builds upon three important assumptions: ① the instrumental variables (IVs) are strongly associated with the exposure, ② the IVs are independent of any confounders, and ③ the IVs affect the outcome only through the exposure but not via other pathways. The blue line represented the Mendelian randomization analysis of the association of OSA/ snoring with AF. The brown line represented the Mendelian randomization analysis of the association of AF with OSA. Abbreviations: OSA, obstructive sleep apnea; AF, atrial fibrillation; IVW (fe/mre) inverse-variance weighted (fixed-effects/multiplicative random-effects); MR-RAPS, MR-robust adjusted profile score; MR-PRESSO, MR-pleiotropy residual sum and outlier; GWAS, genome-wide association study; AF HRC, Atrial Fibrillation Haplotype Reference Consortium

A two-sample bidirectional MR approach was used to estimate the causal inference between OSA and AF. MR approach builds upon three important assumptions: (1) the IVs are strongly associated with the exposure, (2) the IVs are independent of any confounders, and (3) the IVs affect the outcome only through the exposure but not via other pathways (Fig. 1).

### Data sources and IVs selection

Detailed information on the data sources were shown in Table 1. Summary-level data of OSA were retrieved from a recent genome-wide association study (GWAS) published by Strausz et al., containing 16,761 OSA patients and 201,194 cases in the FinnGen Study [12]. Cases of OSA were defined based on the International Classification of Diseases (ICD) codes (ICD-10: G47.3, ICD-9: 3472A) [12]. 5 distinct genetic loci genome-wide significantly associated with OSA (*P* < 5 × 10^–8^) were identified [12]. We implemented the latest GWAS meta-analysis for AF conducted by Nielsen et al. involving six contributing studies, including the Nord-Trøndelag Health Study (HUNT), deCODE, the Michigan Genomics Initiative (MGI), DiscovEHR, UK Biobank, and the AF HRC Consortium in this study [13]. This study, including 60,620 patients with AF (mainly diagnosed according to ICD‐9 or ICD‐10) and 970,216 controls of European ancestry, reported 111 independent genetic loci (*r*^2^ < 0.10) reaching genome-wide significance (*P* < 5 × 10^–8^) [13]. We utilized the reported single nucleotide polymorphisms (SNPs) as IVs for OSA and AF in the present study, and extracted the SNP-outcome associations from the summary statistics for outcomes (Additional file 1: Table S1-2). Those SNPs not available in the outcome datasets were replaced by proxy SNPs (*r*^2^ > 0.8) by searching an online website (http://snipa.helmholtz-muenchen.de/snipa3/).

**Table 1:** Characteristics of data sources used in the Mendelian randomization study

Traits	Data sources	Sample size (cases/controls)	Ancestry	Use
OSA	FinnGen [12]	16,761/201,194	European	Exposure/Outcome
AF	Nielsen et al. [13]	60,620/970,216	European	Exposure/Outcome
AF	AF HRC [14]	65,446/522,744	Mixed (84.2% European)	Outcome for replication analyses
Snoring	UK Biobank [15]	152,302/256,015	European	Additional analyses
Body mass index	Hoffmann et al. [16]	334,487	Mixed (94.3% European)	Confounder for MVMR analyses
HTN, SBP, DBP	Neale laboratory [17]	317,754	European	Confounder for MVMR analyses
Type 2 diabetes	Mahajan et al. [18]	74,124/824,006	European	Confounder for MVMR analyses
LDL-C, HDL-C, TG	Richardson et al. [19]	403,943 ~ 441,016	European	Confounder for MVMR analyses

OSA, obstructive sleep apnea; AF, atrial fibrillation; MVMR, multivariable Mendelian randomization; HTN, hypertension; SBP, systolic blood pressure; DBP, diastolic blood pressure; LDL-C, low-density lipoprotein cholesterol; HDL-C, high-density lipoprotein cholesterol; TG, triglycerides; AF HRC, Atrial Fibrillation Haplotype Reference Consortium

### Statistical analysis

We used the fixed-effects inverse-variance weighted (IVW) model as the main statistical method. Several sensitivity analyses, including the weighted median [20], MR-Egger [21], MR-robust adjusted profile score (MR-RAPS) [22], and the MR-pleiotropy residual sum and outlier (MR-PRESSO) [23], were applied to examine the robustness of the results and possible pleiotropy. The weighted median method assumed that more than 50% of the weight was derived from valid SNPs and could provide consistent estimates on causal effects [20]. The MR-Egger approach provided estimates after correcting for pleiotropic effects, although at the cost of lower statistical power [21]. The MR-RAPS corrected for horizontal pleiotropy in the IVW analysis by using robust adjusted profile scores [22]. The MR-PRESSO method detected and corrected for outliers, providing MR estimates robust to heterogeneity after removing the identified outliers [23]. As pleiotropy in the MR analyses can lead to confounding and bias of MR estimates, investigation of pleiotropy is of importance. We undertook multiple methods to identify potential pleiotropy. First, Cochran's Q statistic was used to assess the heterogeneity among IVs. A Cochran's Q-derived *P* < 0.05 was deemed as a marker of heterogeneity, and in that case, the multiplicative random-effects IVW method would be implemented. Second, the intercept test from the MR-Egger method was conducted to measure for pleiotropy [21]. Third, we performed the leave-one-out analysis to evaluate the consistency of associations and identify whether the results were driven by any individual SNP. Last, we searched each IV in the PhenoScanner database (version 2.0) to evaluate if there were any previous significant associations (*P* < 5 × 10^–8^) with confounders, including body mass index (BMI), weight, blood pressure, and glycemic and lipid traits (Additional file 1: Table S3). We further performed the MR analyses by ruling out potential pleiotropic SNPs.

Replication analyses were performed to validate the reliability of our results by using another GWAS for AF from the AF HRC consortium (Atrial Fibrillation Haplotype Reference Consortium) as the outcome dataset, which consists of 65,446 AF cases and 522,744 controls (Table 1) [14]. Additional analyses were further implemented to evaluate the robustness of the significant causal effect of OSA on AF through estimating the causality of OSA-related phenotype (snoring) with AF. IVs for snoring were obtained from a recent GWAS published by Campos et al. by using UK biobank data (152,302 cases and 256,015 control) (Table 1) [15]. This study identified 41 genome-wide significant loci, and all these 41 SNPs were in different genomic regions and were not in linkage disequilibrium (*r*^2^ < 0.10) [15]. The reported SNPs were used to determine snoring-associated IVs. After excluding 10 non-biallelic SNPs and 1 SNP genome-wide significantly associated with AF in the AF GWAS from Nielsen et al. (rs4744369, *P* = 1.83 × 10^–27^), 30 SNPs remained as IVs (Additional file 1: Table S4).

Previous studies have shown that OSA is strongly correlated with cardiovascular and metabolic traits [12]. Besides, associations between rs9937053 and BMI, type 2 diabetes mellitus (T2D), and hypertension (HTN) were found in the PhenoScanner database. Therefore, we performed regression-based multivariable MR to obtain direct effects of OSA on AF that were independent of potential confounders (BMI, blood pressure, T2D, and circulating lipid levels). We extracted the genetic associations of instruments with those confounders from publicly available GWASs, including BMI [16], blood pressure measurements [HTN, systolic blood pressure (SBP), and diastolic blood pressure (DBP)] [17], T2D [18], and circulating lipid levels [low-density lipoprotein cholesterol (LDL-C), high-density lipoprotein cholesterol (HDL-C), and triglycerides (TG)] [19], respectively. Detailed information for each GWAS was displayed in Table 1.

The reported odds ratios (ORs) and corresponding 95% confidence intervals (CIs) were interpreted per one-unit increase of log odds of exposure traits (OSA, AF, and snoring). Associations with P-values < 0.05 were considered to indicate statistical significance. All analyses were conducted in R foundation (Version 4.0.2) by using package TwosampleMR [24], MR-PRESSO [23], and MendelianRandomization [25].

## Results

### Genetic liability to OSA with AF

**Fig. 2:**
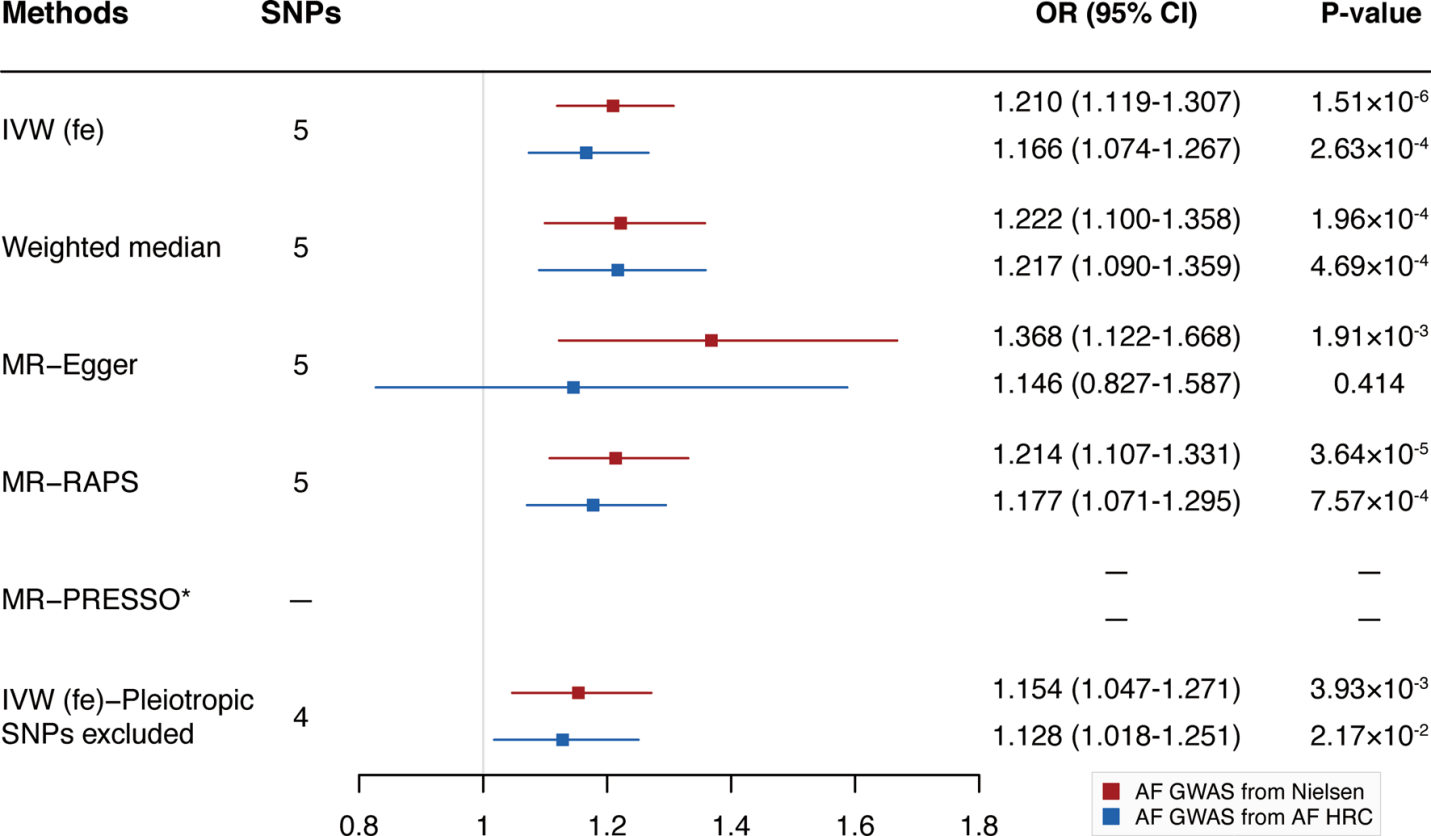
Mendelian randomization estimates of obstructive sleep apnea on atrial fibrillation using two outcome datasets. Abbreviations: MR, Mendelian randomization; SNPs, single nucleotide polymorphisms; OR, odds ratio; CI, confidence interval; IVW (fe), fixed-effects inverse-variance weighted; MR-RAPS, MR-robust adjusted profile score; MR-PRESSO, MR-pleiotropy residual sum and outlier; *No outlier was detected

In the fixed-effects IVW analysis, genetic liability to OSA was associated with a higher risk of AF using the outcome dataset from Nielsen et al. (OR 1.210; 95% CI 1.119–1.307; *P* = 1.51 × 10^–6^) (Fig. 2, Additional file 1: Table S5). There was no presence of heterogeneity estimated by Cochran's Q statistics (*P* = 0.245; Additional file 1: Table S5) or pleiotropy detected by MR-Egger intercept and MR-PRESSO method (P_intercept_ = 0.272, P_MR-PRESSO global test_ = 0.240; Additional file 1: Table S5). Sensitivity analyses, including the weighted median, MR-Egger, and MR-RAPS, yielded robust and consistent results (Fig. 2, Additional file 1: Table S5). Removal of the pleiotropic genetic variant (rs9937053) identified in the PhenoScanner database did not change the significant result (Fig. 2, Additional file 1: Table S5). Furthermore, the significant result was not driven by any single SNP according to the leave-one-out analysis (Additional file 1: Figure S1).

When performing replication analyses using another AF GAWS from AF HRC, the causal association between OSA with AF remained stable and robust except for the MR-Egger approach which had broader CIs (Fig. 2, Additional file 1: Table S5). To further validate the reliability of our results, we used the OSA-related phenotype (snoring) as the exposure. A significant causal association between snoring and AF was observed across all MR methods except for the weighted median and MR-Egger methods, although increased ORs were observed (Additional file 1: Figure S2, Additional file 1: Table S5).

To obtain the direct causal effect of OSA on AF, we used the regression-based multivariable MR approach to correct for potential confounders (BMI, blood pressure, T2D, circulating lipid levels, and snoring). After adjusting for every single confounder, OSA remained to be associated with an increased risk of AF (Table 2).

**Table 2:** Causal effects of obstructive sleep apnea on atrial fibrillation from multivariable Mendelian randomization

Exposure	Obstructive sleep apnea	
Model	OR (95% CI)	P-value
Unadjusted model	1.210 (1.119–1.307)	1.51 × 10^–6^
Adjusted for BMI	1.185 (1.083–1.295)	1.98 × 10^–4^
Adjusted for hypertension	1.221 (1.124–1.327)	2.47 × 10^–6^
Adjusted for SBP	1.237 (1.097–1.395)	5.36 × 10^–4^
Adjusted for DBP	1.210 (1.069–1.369)	2.58 × 10^–3^
Adjusted for T2D	1.146 (1.024–1.283)	1.75 × 10^–2^
Adjusted for LDL-C	1.149 (1.012–1.305)	3.18 × 10^–2^
Adjusted for HDL-C	1.172 (1.024–1.341)	2.09 × 10^–2^
Adjusted for TG	1.284 (1.149–1.435)	1.07 × 10^–5^
Adjusted for snoring	1.216 (1.059–1.397)	5.55 × 10^–3^

The results are presented as odds ratios (95% CIs) for the effect of per one-unit increase of log odds of obstructive sleep apnea

OR, odds ratio; CI, confidence interval; BMI, body mass index; SBP, systolic blood pressure; DBP, diastolic blood pressure; T2D, type 2 diabetes mellitus; LDL-C, low-density lipoprotein cholesterol; HDL-C, high-density lipoprotein cholesterol; TG, triglycerides

### Genetic liability to AF with OSA

**Fig. 3:**
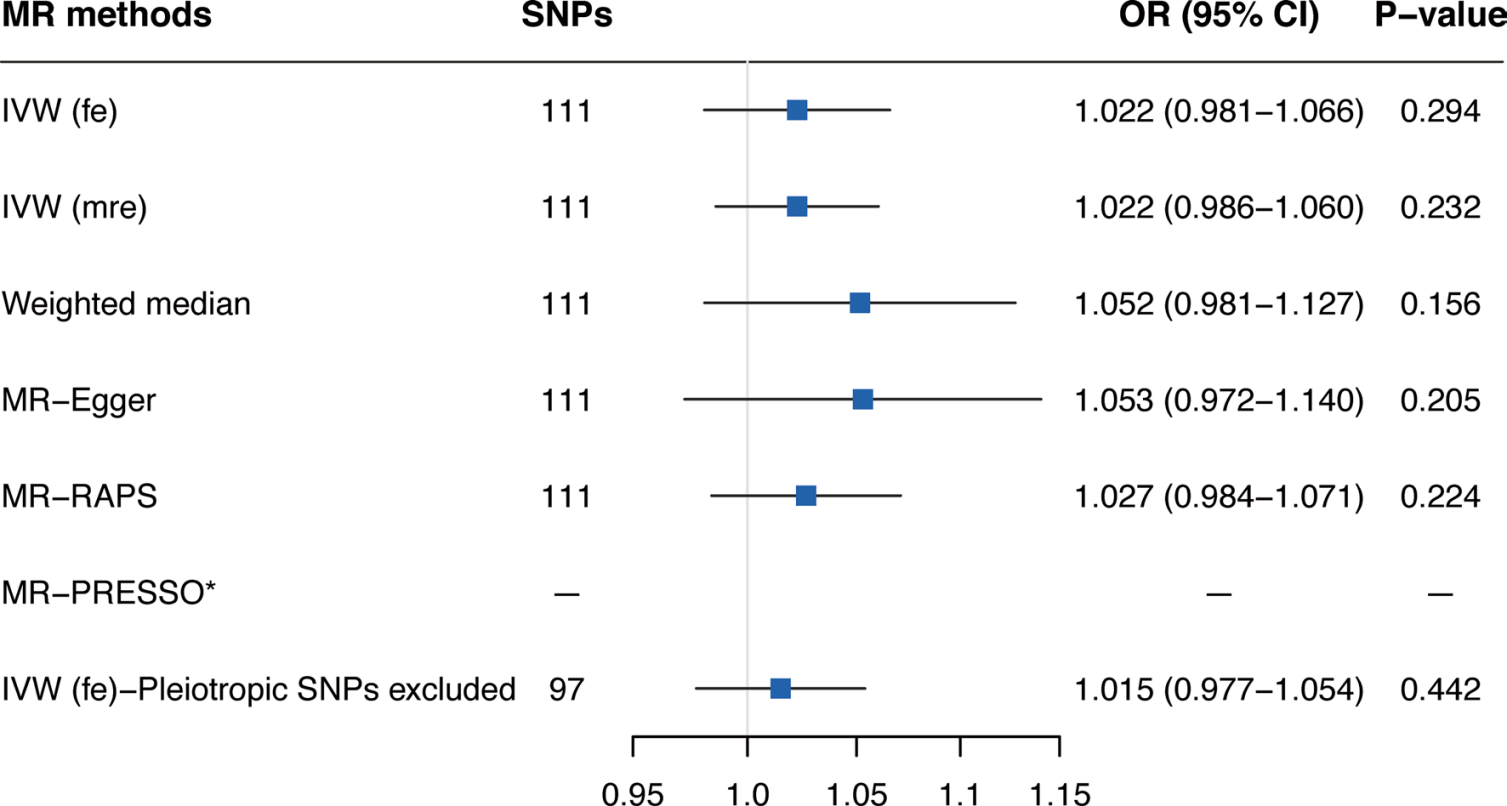
Mendelian randomization estimates of atrial fibrillation on obstructive sleep apnea. Abbreviations: MR, Mendelian randomization; SNPs, single nucleotide polymorphisms; OR, odds ratio; CI, confidence interval; IVW (fe), fixed-effects inverse-variance weighted; IVW (mre), multiplicative random-effects inverse-variance weighted; MR-RAPS, MR-robust adjusted profile score; MR-PRESSO, MR-pleiotropy residual sum and outlier; *No outlier was detected

Reverse MR analyses did not obtain a causal association between genetic liability to AF with OSA using the fixed-effects IVW method (OR 1.022; 95% CI 0.981–1.066; *P* = 0.294; Fig. 3, Additional file 1: Table S5). The lack of association remained in the weighted median method, MR-Egger method, and MR-RAPS analysis (Fig. 3, Additional file 1: Table S5). There was evidence of heterogeneity and horizontal pleiotropy (P for Cochran's Q statistics = 0.019, P_MR-PRESSO global test_ = 0.023; Additional file 1: Table S5); nevertheless, no directional pleiotropy or outlier was identified (P_intercept_ = 0.399; Additional file 1: Table S5). Thus, we moved from the fixed-effects IVW method to the random-effects IVW model, and similarly, there was also no causal association between genetically predicted AF and OSA. The leave-one-out analysis demonstrated that no heterogeneous SNPs largely affected the causal estimates (Additional file 1: Figure S3). Moreover, analyses excluding the 14 pleiotropic SNPs did not change the results (Fig. 3, Additional file 1: Table S5).

## Discussion

This two-sample bidirectional MR study demonstrated that genetic predisposition to OSA was causally associated with an increased risk of AF. On the other hand, there was no evidence supporting a causal association of AF with the risk of OSA.

Previous observational studies have concluded that OSA is a possible independent risk factor for AF. For instance, a large sleep-clinic cohort showed that, after adjusting for multivariate predictors of AF, including age, BMI, diabetes, etc., OSA diagnosis was independently predictive of AF [hazard ratio (HR) 1.55; 95% CI 1.21–2.00; *P* < 0.001] [6]. This study further reported a dose–response correlation between the severity of OSA and the risk of AF, with mild, moderate, and severe OSA associated with 1.48-fold, 1.51-fold, and 1.73-fold higher risk of incident AF, respectively. Another clinic-based study also indicated that OSA was a strong predictor of incident AF (HR 2.18; 95% CI 1.34–3.54; *P* = 0.002), as well as multiple measures of OSA severity [26]. Likewise, in a large arrhythmia-free clinical cohort of 8,256 subjects with suspected OSA, severe nocturnal hypoxemia was found to be independently associated with an increased risk of incident hospitalized AF [27]. However, in a community-based prospective study, Tung et al. found no association between obstructive apnea‐hypopnea index and incident AF [28]. The divergent findings of previous studies might be due to variability in the OSA diagnosis criteria, sample size, and study population. In addition, these observational studies could be limited by selection bias, short follow-up duration, as well as unmeasured confounding factors. Applying a two-sample MR approach, our study provided causal evidence that OSA was a risk factor for AF, which was in line with most previous studies and less susceptible to confounding factors and reverse causation.

Recently, Chen et al. used the MR method to evaluate the causal effect of OSA on AF and found that genetically predicted OSA was associated with an increased risk of AF in both the fixed‐effects and random‐effects IVW models, and the results were robust in sensitivity analyses [29]. Consistently, a significant causal association between OSA and AF was observed in our MR study. However, our study did further investigations to make our results more creditable. First, we applied bidirectional MR design, which additionally found no causal effect of AF on OSA. Second, we replicated the detrimental effect of OSA on AF in another AF data source. In addition, we estimated the causality of OSA-related phenotype (snoring) with AF, and the results also revealed a causal association. Last, we further obtained the direct effect of OSA on AF with a multivariable MR approach, considering OSA is strongly correlated with cardiovascular and metabolic traits. After accounting for each risk factor, the causal effect of OSA on AF risk remained.

Multiple pathophysiological mechanisms might mediate the causal association of OSA with AF risk. Abnormalities of gas exchange in OSA patients, characterized as intermittent hypoxia and hypercapnia, might impact the AF substrate [26, 27]. Moreover, repetitive hypoxia and reoxygenation could lead to oxidative stress and systemic inflammation, which have already been implicated in AF [28]. Sympathetic and parasympathetic imbalances due to OSA also seem to play an essential role in AF pathogenesis [30]. In addition, significant atrial structural and electrical remodeling forming the substrate predisposing to AF was observed in OSA patients, including atrial enlargement, voltage reduction, and conduction abnormalities [31, 32].

As the present MR study indicated that OSA exerted a detrimental impact on AF, early screening and appropriate management of OSA might become an essential issue. It was reported that continuous positive airway pressure (CPAP) therapy in OSA patients significantly prevented the occurrence of paroxysmal AF (*P* < 0.001) [33]. In addition, several meta-analyses concluded that treating OSA with CPAP could improve the effectiveness of AF interventions, reduce the risk of AF recurrence, improve the atrial conduction, and prevent the atrial remodeling [34–36], which further supported the importance of identifying and managing OSA in AF patients.

Prior studies found that the prevalence of OSA in AF patients was much higher than in the general population [4]; however, prospective randomized trials are rare regarding the effect of AF on OSA. Several small-scale studies have shown controversial results of whether restoration of sinus rhythm from AF could reduce the burden of OSA. Naruse et al. reported a reduction in OSA among 25 AF patients after radiofrequency catheter ablation [37], and similarly, Fox et al. found that restoration of sinus rhythm of patients with AF or atrial flutter substantially decreased apnea–hypopnea index (from 23.4 ± 16.3 to 16.3 ± 11.5/h, *P* < 0.001) [38]. Whereas, multiple studies suggested that restoring the atrial function of AF patients did not affect the prevalence and severity of OSA [39–41], eliminating that AF did not play a causative or aggravating role in OSA. Considering the small sample size and short follow-up periods of the existing studies, it is hard to conclude whether AF could affect the risk of OSA. In this MR study, no causality of genetically predicted AF with the risk of OSA was observed, which further supported the evidence that OSA is a risk factor for AF, but not the opposite.

This bidirectional MR study used the latest datasets available for the exposures and outcomes to comprehensively investigate the causal inference between OSA and AF, avoiding possible confounders and reverse causation of traditional observational studies. In addition, a range of sensitivity analyses, replication analyses, and multivariable MR analyses were applied, to test the robustness and reliability of our results. It turned out that the causal estimates in this study were robust and reliable. However, some limitations exist in this study. First, the participants of the datasets involved in our study were mainly from the European-ancestry, which limited the generalization of our results to other populations. Second, although there was no sample overlap between the FinnGen study and two AF datasets, the GWAS for snoring was from the UK Biobank, leading to a small sample overlap with the AF dataset, which might cause minor bias in MR estimates. Third, potential pleiotropy would violate three MR assumptions, which in turn biased our results. Nonetheless, several approaches were performed to minimize the probability of pleiotropy bias, including identifying pleiotropy with MR-Egger intercept analysis, detecting outliers by the MR-PRESSO method, and searching pleiotropic IVs in the PhenoScanner database. Last, we were unable to assess the sexual effects on the associations between OSA and AF because of lacking sex-specific data.

## Conclusion

In conclusion, our MR study suggested that genetic liability to OSA was causally associated with an increased risk of AF. On the other hand, no causal effect of AF on OSA risk was observed. Considering the immense disease burden and the causal link obtained in this study, early screening and appropriate management of OSA might show anti-arrhythmic benefits.

## Supplementary Information

**Additional file 1.** **Table S1.** Characteristics of the genetic variants used for Mendelian randomization analysis of obstructive sleep apnea on atrial fibrillation. **Table S2.** Characteristics of the genetic variants used for Mendelian randomization analysis of atrial fibrillation on obstructive sleep apnea. **Table S3.** Characteristics of the SNPs significantly associated with potential confounders (P < 5×10-8). **Table S4.** Characteristics of the genetic variants used for Mendelian randomization analysis of snoring on atrial fibrillation. **Table S5.** Results of causal inference between obstructive sleep apnea and atrial fibrillation. **Figure S1.** Leave-one-out analysis for causal effect of obstructive sleep apnea on atrial fibrillation. **Figure S2.** Additional Mendelian randomization analyses of the associations of snoring with atrial fibrillation. **Figure S3.** Leave-one-out analysis for causal effect of atrial fibrillation on obstructive sleep apnea.

## Data Availability

Our study used publicly available summary-level data of GWAS. The summary statistics for obstructive sleep apnea are available for download under the phenocode G6_SLEEPAPNO at https://www.finngen.fi/en. The meta-analysis summary association statistics for atrial fibrillation from Nielsen et al. are available through http://csg.sph.umich.edu/willer/public/afib2018. The summary statistics of GWAS for atrial fibrillation from AF HRC are derived from the Cardiovascular Disease Knowledge Portal (http://www.broadcvdi.org/). Full GWAS summary statistics for snoring and body mass index are publicly available through the NHGRI-EBI GWAS Catalogue (https://www.ebi.ac.uk/gwas/downloads/summary-statistics). Summary level data for blood pressure measurements can be accessed at http://www.nealelab.is/uk-biobank/. The data for type 2 diabetes is available at the DIAGRAM consortium website http://diagram-consortium.org/. And summary statistics for circulating lipid levels are available from https://gwas.mrcieu.ac.uk. The code used for this study is openly available on GitHub (https://github.com/Cl-sunny/Mendelian-randomization-OSA-AF.git).

## Abbreviations

AF: Atrial fibrillation
OSA: Obstructive sleep apnea
MR: Mendelian randomization
IVs: Instrumental variables
GWAS: Genome-wide association study
ICD: International Classification of Diseases
SNPs: Single nucleotide polymorphisms
IVW: Inverse-variance weighted
MR-RAPS: MR-robust adjusted profile score
MR-PRESSO: MR-pleiotropy residual sum and outlier
BMI: Body mass index
AF HRC: Atrial Fibrillation Haplotype Reference Consortium
T2D: Type 2 diabetes mellitus
HTN: Hypertension
SBP: Systolic blood pressure
DBP: Diastolic blood pressure
LDL-C: Low-density lipoprotein cholesterol
HDL-C: High-density lipoprotein cholesterol
TG: Triglycerides
ORs: Odds ratios
CIs: Confidence intervals
HR: Hazard ratio
CPAP: Continuous positive airway pressure

## References

[1] Hindricks G (2021). 2020 ESC Guidelines for the diagnosis and management of atrial fibrillation developed in collaboration with the European Association for Cardio-Thoracic Surgery (EACTS): The Task Force for the diagnosis and management of atrial fibrillation of the European Society of Cardiology (ESC) Developed with the special contribution of the European Heart Rhythm Association (EHRA) of the ESC. Eur Heart J.

[2] Rahman F, Kwan GF, Benjamin EJ (2014). Global epidemiology of atrial fibrillation. Nat Rev Cardiol.

[3] Linz D (2015). The importance of sleep-disordered breathing in cardiovascular disease. Clin Res Cardiol.

[4] Linz D (2018). Associations of obstructive sleep apnea with atrial fibrillation and continuous positive airway pressure treatment: a review. JAMA Cardiol.

[5] Mehra R (2006). Association of nocturnal arrhythmias with sleep-disordered breathing: the Sleep Heart Health Study. Am J Respir Crit Care Med.

[6] Cadby G (2015). Severity of OSA is an independent predictor of incident atrial fibrillation hospitalization in a large sleep-clinic cohort. Chest.

[7] Ng CY (2011). Meta-analysis of obstructive sleep apnea as predictor of atrial fibrillation recurrence after catheter ablation. Am J Cardiol.

[8] Monahan K (2012). Relation of the severity of obstructive sleep apnea in response to anti-arrhythmic drugs in patients with atrial fibrillation or atrial flutter. Am J Cardiol.

[9] Zhang L, Hou Y, Po SS (2015). Obstructive sleep apnoea and atrial fibrillation. Arrhythm Electrophysiol Rev.

[10] Gami AS (2004). Association of atrial fibrillation and obstructive sleep apnea. Circulation.

[11] Sekula P (2016). Mendelian randomization as an approach to assess causality using observational data. J Am Soc Nephrol.

[12] Strausz S (2021). Genetic analysis of obstructive sleep apnoea discovers a strong association with cardiometabolic health. Eur Respir J.

[13] Nielsen JB (2018). Biobank-driven genomic discovery yields new insight into atrial fibrillation biology. Nat Genet.

[14] Roselli C (2018). Multi-ethnic genome-wide association study for atrial fibrillation. Nat Genet.

[15] Campos AI (2020). Insights into the aetiology of snoring from observational and genetic investigations in the UK Biobank. Nat Commun.

[16] Hoffmann TJ (2018). A large multiethnic genome-wide association study of adult body mass index identifies novel loci. Genetics.

[17] Neale Lab. UK Biobank GWAS results. 2018. http://www.nealelab.is/uk-biobank/. Accessed 25 April 2021.

[18] Mahajan A (2018). Fine-mapping type 2 diabetes loci to single-variant resolution using high-density imputation and islet-specific epigenome maps. Nat Genet.

[19] Richardson TG (2020). Evaluating the relationship between circulating lipoprotein lipids and apolipoproteins with risk of coronary heart disease: a multivariable Mendelian randomisation analysis. PLoS Med.

[20] Bowden J (2016). Consistent estimation in mendelian randomization with some invalid instruments using a weighted median estimator. Genet Epidemiol.

[21] Bowden J, Davey Smith G, Burgess S (2015). Mendelian randomization with invalid instruments: effect estimation and bias detection through Egger regression. Int J Epidemiol.

[22] Zhao Q (2018). Statistical inference in two-sample summary-data Mendelian randomization using robust adjusted profile score. Ann Stat.

[23] Verbanck M (2018). Detection of widespread horizontal pleiotropy in causal relationships inferred from Mendelian randomization between complex traits and diseases. Nat Genet.

[24] Hemani G (2018). The MR-Base platform supports systematic causal inference across the human phenome. Elife.

[25] Yavorska OO, Burgess S (2017). MendelianRandomization: an R package for performing Mendelian randomization analyses using summarized data. Int J Epidemiol.

[26] Gami AS (2007). Obstructive sleep apnea, obesity, and the risk of incident atrial fibrillation. J Am Coll Cardiol.

[27] Nalliah CJ, Sanders P, Kalman JM (2016). Obstructive sleep apnea treatment and atrial fibrillation: a need for definitive evidence. J Cardiovasc Electrophysiol.

[28] May AM, Van Wagoner DR, Mehra R (2017). OSA and cardiac arrhythmogenesis: mechanistic insights. Chest.

[29] Chen W, Cai X, Yan H, Pan Y. Causal effect of obstructive sleep apnea on atrial fibrillation: a mendelian randomization study. J Am Heart Assoc. 2021;10(23):e022560. 10.1161/JAHA.121.022560 PMC9075405 34796736

[30] Baranchuk A (2008). It's time to wake up! Sleep apnea and cardiac arrhythmias. Europace.

[31] Dimitri H (2012). Atrial remodeling in obstructive sleep apnea: implications for atrial fibrillation. Heart Rhythm.

[32] Anter E (2017). Atrial substrate and triggers of paroxysmal atrial fibrillation in patients with obstructive sleep apnea. Circ Arrhythm Electrophysiol.

[33] Abe H (2010). Efficacy of continuous positive airway pressure on arrhythmias in obstructive sleep apnea patients. Heart Vessels.

[34] Shukla A (2015). Effect of obstructive sleep apnea treatment on atrial fibrillation recurrence: a meta-analysis. JACC Clin Electrophysiol.

[35] Qureshi WT (2015). Meta-analysis of continuous positive airway pressure as a therapy of atrial fibrillation in obstructive sleep apnea. Am J Cardiol.

[36] Lavergne F (2015). Atrial fibrillation and sleep-disordered breathing. J Thorac Dis.

[37] Naruse Y (2012). Radiofrequency catheter ablation of persistent atrial fibrillation decreases a sleep-disordered breathing parameter during a short follow-up period. Circ J.

[38] Fox H (2016). Cardioversion of atrial fibrillation or atrial flutter into sinus rhythm reduces nocturnal central respiratory events and unmasks obstructive sleep apnoea. Clin Res Cardiol.

[39] Höglund N (2017). Cardioversion of atrial fibrillation does not affect obstructive sleep apnea. Ups J Med Sci.

[40] Hoyer FF (2014). Impact of pulmonary vein isolation on obstructive sleep apnea in patients with atrial fibrillation. Cardiol J.

[41] Lissel C (2008). Effect of restoring sinus rhythm on sleep apnea in patients with atrial fibrillation or flutter. Am J Cardiol.

