# Supplementary material for: Obstructive sleep apnea and atrial fibrillation: insights from a bidirectional Mendelian randomization study
Source: BMC Med Genomics. 2022 Feb 16;15:28. doi: 10.1186/s12920-022-01180-5 (PMC8851818; doi:10.1186/s12920-022-01180-5)
Supplement: Supplementary file 1 — Additional file 1. Table S1. Characteristics of the genetic variants used for Mendelian randomization analysis of obstructive sleep apnea on atrial fibrillation. Table S2. Characteristics of the genetic variants used for Mendelian randomization analysis of atrial fibrillation on obstructive sleep apnea. Table S3. Characteristics of the SNPs significantly associated with potential confounders (P < 5×10^-8^). Table S4. Characteristics of the genetic variants used for Mendelian randomization analysis of snoring on atrial fibrillation. Table S5. Results of causal inference between obstructive sleep apnea and atrial fibrillation. Figure S1. Leave-one-out analysis for causal effect of obstructive sleep apnea on atrial fibrillation. Figure S2. Additional Mendelian randomization analyses of the associations of snoring with atrial fibrillation. Figure S3. Leave-one-out analysis for causal effect of atrial fibrillation on obstructive sleep apnea. [file 12920_2022_1180_MOESM1_ESM.docx]

# Supplemental Material

# ****Supplementary Table****

**Table S1.** Characteristics of the genetic variants used for Mendelian randomization analysis of obstructive sleep apnea on atrial fibrillation.

| **SNP** | **Chromosome position** | **Nearest Gene** | **Alleles (E/A)** | **EAF** | **R^2^** | **F** | **SNP-OSA Associations** | | | **SNP-AF Associations (Nielsen)** | | | **SNP-AF Associations (AF HRC)** | | |
| --- | --- | --- | --- | --- | --- | --- | --- | --- | --- | --- | --- | --- | --- | --- | --- |
|  |  |  |  |  |  |  | **Beta** | **SE** | **P value** | **Beta** | **SE** | **P value** | **Beta** | **SE** | **P value** |
| rs9937053 | 16:53765595 | FTO | A/G | 0.430 | 5.10E-03 | 1117.1 | 0.102 | 0.013 | 4.32E-16 | 0.028 | 0.007 | 3.05E-05 | 0.022 | 0.007 | 2.63E-03 |
| rs10507084 | 12:97359374 | NEDD1 | T/C | 0.179 | 3.46E-03 | 757.7 | 0.109 | 0.016 | 2.80E-11 | 0.011 | 0.013 | 4.19E-01 | -0.009 | 0.013 | 5.09E-01 |
| rs4837016 | 9:125379530 | GAPVD1 | A/G | 0.466 | 2.48E-03 | 542.0 | -0.071 | 0.013 | 1.53E-08 | -0.003 | 0.007 | 6.12E-01 | -0.019 | 0.007 | 8.12E-03 |
| rs185932673 | 10:12656440 | CAMK1D | T/C | 0.003 | 2.58E-03 | 562.8 | 0.624 | 0.112 | 2.44E-08 | 0.161 | 0.054 | 2.92E-03 | 0.117 | 0.063 | 6.40E-02 |
| rs10928560 | 2:136234237 | CXCR4 | T/C | 0.195 | 2.42E-03 | 528.6 | -0.088 | 0.016 | 2.80E-08 | -0.011 | 0.009 | 2.27E-01 | -0.004 | 0.009 | 6.45E-01 |

SNP, single nucleotide polymorphism; E/A, effect/alternative alleles; EAF, effect allele frequency; F, the F statistic for each SNP, was calculated as: F=R^2^×(n-2)/(1-R^2^); OSA, obstructive sleep apnea; AF, atrial fibrillation; SE, standard error; AF HRC, Atrial Fibrillation Haplotype Reference Consortium.

**Table S2.** Characteristics of the genetic variants used for Mendelian randomization analysis of atrial fibrillation on obstructive sleep apnea.

| **SNP** | **Chromosome position** | **Nearest Gene** | **Alleles (E/A)** | **EAF** | **R^2^** | **F** | **SNP-AF Associations (Nielsen)** | | | **SNP-OSA Associations** | | |
| --- | --- | --- | --- | --- | --- | --- | --- | --- | --- | --- | --- | --- |
|  |  |  |  |  |  |  | **Beta** | **SE** | **P value** | **Beta** | **SE** | **P value** |
| rs67249485 | 4:111699685 | PITX2 | T/A | 0.199 | 1.97E-03 | 2036.1 | 0.366 | 0.008 | 7.32E-443 | 0.019 | 0.015 | 1.87E-01 |
| rs2359171 | 16:73053022 | ZFHX3 | A/T | 0.176 | 4.00E-04 | 412.2 | 0.175 | 0.009 | 4.65E-91 | 0.021 | 0.015 | 1.58E-01 |
| rs11264280 | 1:154862952 | KCNN3 | T/C | 0.333 | 3.49E-04 | 359.9 | 0.135 | 0.007 | 3.07E-79 | 0.021 | 0.013 | 1.06E-01 |
| rs11598047 | 10:105342672 | NEURL1 | G/A | 0.162 | 2.83E-04 | 291.6 | 0.154 | 0.009 | 8.95E-66 | 0.019 | 0.021 | 3.82E-01 |
| rs11773845 | 7:116191301 | CAV1, CAV2 | A/C | 0.586 | 2.40E-04 | 247.5 | 0.105 | 0.007 | 2.39E-55 | -0.001 | 0.013 | 9.45E-01 |
| rs72700114 | 1:170193825 | LINC01142 | C/G | 0.076 | 2.34E-04 | 241.7 | 0.202 | 0.013 | 3.29E-54 | -0.009 | 0.028 | 7.53E-01 |
| rs883079 | 12:114793240 | TBX5 | T/C | 0.707 | 1.70E-04 | 175.7 | 0.098 | 0.007 | 2.84E-40 | -0.016 | 0.013 | 2.47E-01 |
| rs74022964 | 15:73677264 | HCN4 | T/C | 0.157 | 1.53E-04 | 158.2 | 0.113 | 0.009 | 3.51E-36 | 0.001 | 0.015 | 9.45E-01 |
| rs2040862 | 5:137419989 | WNT8A, NPY6R, MYOT, FAM13B | T/C | 0.178 | 1.51E-04 | 155.2 | 0.108 | 0.009 | 1.08E-35 | 0.013 | 0.016 | 4.08E-01 |
| rs60212594 | 10:75414344 | SYNPO2L, NUDT13, MYOZ1, AGAP5 | C/G | 0.144 | 1.46E-04 | 150.1 | -0.118 | 0.010 | 9.20E-35 | -0.023 | 0.017 | 1.89E-01 |
| rs10821415 | 9:97713459 | C9orf3 | A/C | 0.413 | 1.46E-04 | 150.2 | 0.082 | 0.007 | 2.92E-34 | -0.029 | 0.013 | 2.65E-02 |
| rs2738413 | 14:64679960 | SYNE2, MIR548AZ, ESR2, MTHFD1 | G/A | 0.505 | 1.31E-04 | 134.8 | -0.078 | 0.007 | 2.55E-31 | 0.011 | 0.013 | 3.96E-01 |
| rs73366713 | 6:16415751 | ATXN1 | A/G | 0.140 | 1.06E-04 | 109.3 | -0.104 | 0.010 | 1.53E-25 | -0.008 | 0.020 | 6.78E-01 |
| rs4963776 | 12:24779491 | LINC00477 | T/G | 0.182 | 1.04E-04 | 107.6 | -0.091 | 0.009 | 1.84E-25 | 0.012 | 0.019 | 5.28E-01 |
| rs2288327 | 2:179411665 | TTN, MIR548N, FKBP7, TTN-AS1 | G/A | 0.156 | 1.03E-04 | 106.6 | 0.092 | 0.009 | 7.26E-25 | -0.032 | 0.017 | 5.77E-02 |
| rs117984853 | 6:149399100 | UST | T/G | 0.101 | 1.02E-04 | 104.7 | 0.123 | 0.012 | 1.34E-24 | 0.001 | 0.019 | 9.75E-01 |
| rs7650482 | 3:12841804 | CAND2 | G/A | 0.640 | 1.00E-04 | 103.2 | 0.071 | 0.007 | 1.79E-24 | 0.008 | 0.013 | 5.08E-01 |
| rs3820888 | 2:201180023 | SPATS2L | C/T | 0.392 | 9.81E-05 | 101.2 | 0.068 | 0.007 | 5.75E-24 | -0.024 | 0.013 | 5.76E-02 |
| rs3951016 | 6:118559658 | SLC35F1, PLN | A/T | 0.459 | 9.07E-05 | 93.5 | 0.065 | 0.007 | 2.15E-22 | -0.013 | 0.012 | 3.04E-01 |
| rs2540949 | 2:65284231 | CEP68 | T/A | 0.385 | 9.11E-05 | 93.9 | -0.066 | 0.007 | 2.95E-22 | 0.001 | 0.013 | 9.59E-01 |
| rs6891790 | 5:172670745 | NKX2-5 | T/G | 0.283 | 8.92E-05 | 92.0 | -0.073 | 0.008 | 4.53E-22 | 0.011 | 0.013 | 4.11E-01 |
| rs7508 | 8:17913970 | ASAH1 | A/G | 0.711 | 8.72E-05 | 89.9 | 0.071 | 0.008 | 1.69E-21 | 0.000 | 0.014 | 9.82E-01 |
| rs11156751 | 14:32990437 | AKAP6 | C/T | 0.285 | 8.46E-05 | 87.2 | 0.072 | 0.008 | 6.94E-21 | -0.012 | 0.013 | 3.45E-01 |
| rs76097649 | 11:128764570 | KCNJ5 | A/G | 0.093 | 8.36E-05 | 86.2 | 0.115 | 0.012 | 1.26E-20 | 0.026 | 0.021 | 2.16E-01 |
| rs10741807 | 11:20011445 | NAV2 | C/T | 0.755 | 8.26E-05 | 85.2 | -0.073 | 0.008 | 1.59E-20 | 0.024 | 0.016 | 1.24E-01 |
| rs6790396 | 3:38771925 | SCN10A, SCN5A | G/C | 0.596 | 8.25E-05 | 85.0 | 0.063 | 0.007 | 2.40E-20 | 0.020 | 0.013 | 1.15E-01 |
| rs10753933 | 1:203026214 | PPFIA4 | G/T | 0.552 | 8.01E-05 | 82.6 | -0.061 | 0.007 | 9.84E-20 | 0.033 | 0.013 | 1.16E-02 |
| rs34969716 | 6:18210109 | KDM1B, DEK | A/G | 0.305 | 7.86E-05 | 81.0 | 0.070 | 0.008 | 1.60E-19 | -0.015 | 0.013 | 2.64E-01 |
| rs13195459 | 6:122403559 | HSF2 | A/G | 0.362 | 7.68E-05 | 79.2 | -0.062 | 0.007 | 4.15E-19 | 0.010 | 0.014 | 4.64E-01 |
| rs62521286 | 8:124551975 | FBXO32 | G/A | 0.066 | 7.69E-05 | 79.3 | 0.120 | 0.014 | 4.50E-19 | 0.019 | 0.024 | 4.43E-01 |
| rs56181519 | 2:175555714 | WIPF1 | T/C | 0.268 | 7.17E-05 | 73.9 | -0.066 | 0.008 | 6.46E-18 | -0.044 | 0.015 | 3.25E-03 |
| rs6580277 | 5:142818123 | NR3C1 | G/A | 0.237 | 6.98E-05 | 71.9 | 0.067 | 0.008 | 1.64E-17 | 0.003 | 0.015 | 8.59E-01 |
| rs2834618 | 21:36119111 | LINC01426 | G/T | 0.106 | 6.89E-05 | 71.0 | -0.094 | 0.011 | 3.41E-17 | 0.001 | 0.020 | 9.79E-01 |
| rs6747542 | 2:70106832 | GMCL1, ANXA4 | C/T | 0.464 | 6.63E-05 | 68.4 | -0.055 | 0.007 | 1.10E-16 | 0.002 | 0.013 | 8.93E-01 |
| rs337705 | 5:113737062 | KCNN2 | G/T | 0.375 | 6.67E-05 | 68.8 | 0.056 | 0.007 | 1.63E-16 | -0.004 | 0.013 | 7.70E-01 |
| rs12908004 | 15:80676925 | ARNT2 | G/A | 0.164 | 6.42E-05 | 66.2 | 0.073 | 0.009 | 4.12E-16 | 0.035 | 0.016 | 2.62E-02 |
| rs10804493 | 3:111554426 | PHLDB2, PLCXD2 | A/G | 0.651 | 6.16E-05 | 63.5 | 0.056 | 0.007 | 1.63E-15 | 0.005 | 0.013 | 7.17E-01 |
| rs73241997 | 14:35173775 | CFL2 | T/C | 0.142 | 6.03E-05 | 62.1 | 0.073 | 0.009 | 2.94E-15 | 0.015 | 0.019 | 4.30E-01 |
| rs146518726 | 1:51535039 | MIR6500 | A/G | 0.033 | 5.83E-05 | 60.1 | 0.161 | 0.021 | 8.27E-15 | 0.011 | 0.035 | 7.55E-01 |
| rs1545300 | 1:112464004 | KCND3 | T/C | 0.309 | 5.67E-05 | 58.4 | -0.056 | 0.007 | 1.48E-14 | -0.008 | 0.014 | 5.85E-01 |
| rs140185678 | 16:2003016 | RPL3L | A/G | 0.035 | 5.62E-05 | 57.9 | 0.166 | 0.022 | 2.43E-14 | -0.050 | 0.030 | 9.74E-02 |
| rs12809354 | 12:32978437 | PKP2 | C/T | 0.144 | 5.66E-05 | 58.3 | 0.072 | 0.009 | 2.89E-14 | 0.033 | 0.018 | 6.65E-02 |
| rs79187193 | 1:147255831 | GJA5 | A/G | 0.057 | 5.60E-05 | 57.7 | -0.116 | 0.015 | 3.15E-14 | 0.009 | 0.027 | 7.52E-01 |
| rs3176326 | 6:36647289 | CDKN1A, PANDAR, PI16 | A/G | 0.198 | 5.26E-05 | 54.2 | -0.063 | 0.009 | 1.42E-13 | 0.038 | 0.016 | 2.11E-02 |
| rs71454237 | 12:70013415 | LRRC10 | A/G | 0.209 | 5.28E-05 | 54.5 | -0.062 | 0.008 | 1.78E-13 | 0.010 | 0.016 | 5.46E-01 |
| rs6829664 | 4:114448656 | CAMK2D | G/A | 0.262 | 5.19E-05 | 53.5 | 0.056 | 0.008 | 1.92E-13 | 0.004 | 0.014 | 7.62E-01 |
| rs12648245 | 4:174641184 | HAND2, HAND2-AS1 | C/T | 0.076 | 5.16E-05 | 53.2 | -0.093 | 0.013 | 3.45E-13 | -0.015 | 0.017 | 3.79E-01 |
| rs4073778 | 1:116297758 | CASQ2 | A/C | 0.564 | 5.10E-05 | 52.6 | 0.049 | 0.007 | 4.96E-13 | 0.023 | 0.013 | 7.07E-02 |
| rs2860482 | 12:57105938 | NACA | C/A | 0.726 | 4.90E-05 | 50.5 | -0.054 | 0.008 | 1.21E-12 | -0.032 | 0.016 | 4.70E-02 |
| rs464901 | 22:18597502 | TUBA8 | C/T | 0.335 | 4.83E-05 | 49.8 | -0.051 | 0.007 | 1.53E-12 | -0.009 | 0.013 | 4.93E-01 |
| rs12245149 | 10:65321147 | REEP3, NRBF2 | A/C | 0.474 | 4.77E-05 | 49.2 | -0.047 | 0.007 | 1.66E-12 | 0.000 | 0.013 | 9.74E-01 |
| rs56201652 | 7:92278116 | CDK6 | A/G | 0.267 | 4.86E-05 | 50.1 | -0.053 | 0.008 | 1.74E-12 | 0.014 | 0.014 | 3.17E-01 |
| rs55734480 | 7:14372009 | DGKB | A/G | 0.249 | 4.79E-05 | 49.4 | 0.055 | 0.008 | 2.20E-12 | -0.017 | 0.015 | 2.49E-01 |
| rs7578393 | 2:26165528 | KIF3C | T/C | 0.796 | 4.72E-05 | 48.7 | 0.061 | 0.009 | 2.42E-12 | -0.018 | 0.014 | 1.87E-01 |
| rs1563304 | 17:44874453 | WNT3 | T/C | 0.178 | 4.75E-05 | 49.0 | 0.064 | 0.009 | 2.56E-12 | -0.018 | 0.019 | 3.45E-01 |
| rs17380837 | 12:26345526 | SSPN | T/C | 0.307 | 4.70E-05 | 48.4 | -0.050 | 0.007 | 4.80E-12 | -0.034 | 0.013 | 8.09E-03 |
| rs72811294 | 17:12618680 | MYOCD | C/G | 0.113 | 4.48E-05 | 46.1 | -0.072 | 0.011 | 9.67E-12 | 0.004 | 0.022 | 8.53E-01 |
| rs35544454 | 2:213266003 | ERBB4 | T/A | 0.192 | 4.45E-05 | 45.8 | -0.059 | 0.009 | 1.10E-11 | -0.002 | 0.015 | 9.10E-01 |
| rs10213171 | 4:148937537 | ARHGAP10 | G/C | 0.061 | 4.47E-05 | 46.1 | 0.091 | 0.013 | 1.32E-11 | -0.005 | 0.021 | 7.94E-01 |
| rs9953366 | 18:46474192 | SMAD7 | C/T | 0.663 | 4.37E-05 | 45.1 | 0.049 | 0.007 | 1.82E-11 | 0.001 | 0.013 | 9.13E-01 |
| rs7789146 | 7:150661409 | KCNH2 | A/G | 0.179 | 4.37E-05 | 45.1 | -0.058 | 0.009 | 2.12E-11 | -0.023 | 0.015 | 1.26E-01 |
| rs72926475 | 2:86594487 | REEP1 | A/G | 0.123 | 4.35E-05 | 44.8 | -0.068 | 0.010 | 2.37E-11 | -0.023 | 0.021 | 2.80E-01 |
| rs6771054 | 3:89489529 | EPHA3 | C/T | 0.404 | 4.38E-05 | 45.2 | -0.046 | 0.007 | 2.42E-11 | -0.003 | 0.013 | 7.87E-01 |
| rs11658278 | 17:38031164 | ZPBP2, GSDMB, ORMDL3 | C/T | 0.521 | 4.24E-05 | 43.7 | -0.044 | 0.007 | 3.46E-11 | 0.016 | 0.013 | 1.94E-01 |
| rs28387148 | 2:127433465 | GYPC | T/C | 0.105 | 4.17E-05 | 43.0 | 0.074 | 0.011 | 6.25E-11 | 0.020 | 0.022 | 3.57E-01 |
| rs4965430 | 15:99268850 | IGF1R | G/C | 0.614 | 3.96E-05 | 40.8 | -0.044 | 0.007 | 1.26E-10 | -0.014 | 0.013 | 2.82E-01 |
| rs34080181 | 3:66454191 | LRIG1, SLC25A26 | A/G | 0.379 | 4.05E-05 | 41.8 | -0.045 | 0.007 | 1.28E-10 | -0.001 | 0.013 | 9.70E-01 |
| rs2031522 | 6:87821501 | CGA | G/A | 0.376 | 3.99E-05 | 41.1 | -0.044 | 0.007 | 1.47E-10 | 0.018 | 0.013 | 1.53E-01 |
| rs2274115 | 9:139094773 | LHX3 | G/A | 0.700 | 3.98E-05 | 41.1 | 0.049 | 0.008 | 1.69E-10 | -0.010 | 0.014 | 4.76E-01 |
| rs147301839 | 15:57924714 | GCOM1/MYZAP | C/A | 0.007 | 3.93E-05 | 40.5 | 0.333 | 0.052 | 1.93E-10 | 0.006 | 0.058 | 9.25E-01 |
| rs7529220 | 1:22282619 | HSPG2 | C/T | 0.847 | 3.90E-05 | 40.2 | 0.062 | 0.010 | 1.98E-10 | 0.020 | 0.015 | 1.86E-01 |
| rs2885697 | 1:41544279 | SCMH1 | T/G | 0.648 | 3.82E-05 | 39.3 | -0.044 | 0.007 | 2.88E-10 | -0.010 | 0.013 | 4.41E-01 |
| rs35005436 | 7:74134911 | GTF2I, LOC101926943, GTF2IRD2 | C/T | 0.155 | 3.86E-05 | 39.8 | 0.061 | 0.010 | 3.34E-10 | 0.037 | 0.020 | 6.10E-02 |
| rs74884082 | 14:73249419 | DPF3 | T/C | 0.250 | 3.88E-05 | 39.9 | -0.049 | 0.008 | 3.48E-10 | 0.013 | 0.018 | 4.71E-01 |
| rs7834729 | 8:21821778 | XPO7 | T/G | 0.115 | 3.82E-05 | 39.4 | -0.065 | 0.010 | 3.55E-10 | 0.002 | 0.022 | 9.19E-01 |
| rs422068 | 14:23864804 | MYH6, MYH7 | C/T | 0.349 | 3.82E-05 | 39.3 | 0.044 | 0.007 | 3.87E-10 | 0.010 | 0.014 | 4.88E-01 |
| rs2012809 | 5:128190363 | SLC27A6 | G/A | 0.790 | 3.72E-05 | 38.3 | 0.058 | 0.009 | 4.92E-10 | 0.019 | 0.016 | 2.14E-01 |
| rs10458660 | 10:77936576 | C10orf11 | G/A | 0.173 | 3.70E-05 | 38.1 | 0.054 | 0.009 | 6.78E-10 | -0.019 | 0.017 | 2.87E-01 |
| rs6462079 | 7:28415827 | CREB5 | A/G | 0.721 | 3.65E-05 | 37.6 | 0.047 | 0.008 | 8.79E-10 | -0.010 | 0.016 | 5.31E-01 |
| rs133902 | 22:26164079 | MYO18B | T/C | 0.427 | 3.68E-05 | 38.0 | 0.042 | 0.007 | 9.14E-10 | -0.003 | 0.013 | 8.10E-01 |
| rs6994744 | 8:141740868 | PTK2 | C/A | 0.495 | 3.65E-05 | 37.7 | 0.041 | 0.007 | 1.09E-09 | 0.003 | 0.013 | 8.44E-01 |
| rs284277 | 1:10790797 | CASZ1 | A/C | 0.617 | 3.63E-05 | 37.4 | -0.042 | 0.007 | 1.25E-09 | -0.015 | 0.013 | 2.43E-01 |
| rs73041705 | 3:24463235 | THRB | C/T | 0.299 | 3.57E-05 | 36.8 | -0.044 | 0.007 | 1.55E-09 | -0.009 | 0.014 | 5.29E-01 |
| rs1458038 | 4:81164723 | FGF5 | T/C | 0.309 | 3.52E-05 | 36.3 | 0.043 | 0.007 | 1.74E-09 | 0.001 | 0.013 | 9.38E-01 |
| rs9899183 | 17:7452977 | TNFSF12, TNFSF12-TNFSF13, SOX15, FXR2 | T/C | 0.714 | 3.52E-05 | 36.3 | 0.045 | 0.008 | 2.02E-09 | 0.030 | 0.015 | 4.25E-02 |
| rs12188351 | 5:168386089 | SLIT3 | A/G | 0.056 | 3.45E-05 | 35.6 | 0.087 | 0.015 | 2.52E-09 | -0.048 | 0.029 | 1.01E-01 |
| rs17005647 | 3:69406181 | FRMD4B | T/C | 0.364 | 3.48E-05 | 35.8 | 0.041 | 0.007 | 2.70E-09 | 0.008 | 0.013 | 5.42E-01 |
| rs9506925 | 13:23368943 | LINC00540, LINC00621, SGCG | T/C | 0.267 | 3.48E-05 | 35.8 | 0.045 | 0.008 | 2.72E-09 | 0.002 | 0.015 | 8.91E-01 |
| rs6596717 | 5:106427609 | LOC102467213 | A/C | 0.605 | 3.42E-05 | 35.3 | -0.040 | 0.007 | 3.00E-09 | -0.008 | 0.013 | 5.27E-01 |
| rs7225165 | 17:1309850 | YWHAE, CRK, MYO1C | A/G | 0.113 | 3.38E-05 | 34.8 | -0.066 | 0.011 | 3.20E-09 | -0.004 | 0.020 | 8.63E-01 |
| rs11590635 | 1:49309764 | AGBL4 | A/G | 0.024 | 3.34E-05 | 34.5 | 0.146 | 0.025 | 4.12E-09 | 0.031 | 0.043 | 4.73E-01 |
| rs7612445 | 3:179172979 | GNB4 | T/G | 0.188 | 3.34E-05 | 34.4 | 0.049 | 0.008 | 4.81E-09 | -0.027 | 0.020 | 1.73E-01 |
| rs4935786 | 11:121661507 | SORL1 | A/T | 0.733 | 3.33E-05 | 34.3 | -0.046 | 0.008 | 4.85E-09 | 0.026 | 0.014 | 6.48E-02 |
| rs12426679 | 12:76237987 | PHLDA1 | T/C | 0.528 | 3.30E-05 | 34.1 | -0.039 | 0.007 | 4.95E-09 | 0.026 | 0.013 | 4.14E-02 |
| rs35620480 | 8:11499908 | GATA4 | C/A | 0.157 | 3.34E-05 | 34.5 | 0.054 | 0.009 | 5.15E-09 | -0.008 | 0.020 | 7.09E-01 |
| rs55985730 | 7:128417044 | OPN1SW, CALU | G/T | 0.060 | 3.28E-05 | 33.9 | 0.087 | 0.015 | 5.24E-09 | 0.004 | 0.023 | 8.77E-01 |
| rs11125871 | 2:61470126 | USP34 | T/C | 0.395 | 3.26E-05 | 33.6 | -0.039 | 0.007 | 6.42E-09 | -0.024 | 0.013 | 6.56E-02 |
| rs10873298 | 14:77426525 | IRF2BPL | T/C | 0.634 | 3.28E-05 | 33.8 | -0.040 | 0.007 | 7.07E-09 | 0.013 | 0.013 | 3.20E-01 |
| rs1278493 | 3:135814009 | PPP2R3A | A/G | 0.565 | 3.17E-05 | 32.7 | -0.039 | 0.007 | 8.77E-09 | 0.004 | 0.013 | 7.75E-01 |
| rs10749053 | 10:112576695 | RBM20 | C/T | 0.842 | 3.18E-05 | 32.7 | -0.056 | 0.010 | 1.05E-08 | -0.003 | 0.019 | 8.94E-01 |
| rs35569628 | 13:113872712 | CUL4A | C/T | 0.223 | 3.10E-05 | 31.9 | -0.045 | 0.008 | 1.38E-08 | 0.003 | 0.014 | 8.30E-01 |
| rs6560886 | 12:133150210 | FBRSL1 | C/T | 0.788 | 3.11E-05 | 32.1 | 0.051 | 0.009 | 1.49E-08 | -0.010 | 0.017 | 5.40E-01 |
| rs67969609 | 2:145760353 | TEX41 | G/C | 0.071 | 3.09E-05 | 31.8 | 0.071 | 0.013 | 1.71E-08 | -0.044 | 0.027 | 1.04E-01 |
| rs60902112 | 3:194800853 | XXYLT1 | T/C | 0.226 | 3.08E-05 | 31.7 | 0.045 | 0.008 | 1.72E-08 | -0.006 | 0.016 | 7.03E-01 |
| rs4951258 | 1:205691316 | NUCKS1, SLC41A1 | A/G | 0.416 | 3.06E-05 | 31.5 | 0.038 | 0.007 | 2.10E-08 | 0.005 | 0.013 | 6.86E-01 |
| rs10773657 | 12:123327900 | HIP1R | A/C | 0.862 | 3.02E-05 | 31.2 | -0.058 | 0.010 | 2.54E-08 | -0.017 | 0.016 | 3.07E-01 |
| rs12604076 | 17:76773638 | CYTH1, USP36 | C/T | 0.522 | 2.97E-05 | 30.6 | -0.037 | 0.007 | 3.63E-08 | -0.022 | 0.013 | 8.90E-02 |
| rs10006327 | 4:103890980 | SLC9B1 | C/T | 0.490 | 2.86E-05 | 29.5 | 0.036 | 0.007 | 4.42E-08 | -0.019 | 0.013 | 1.24E-01 |
| rs8088085 | 18:48708548 | MEX3C | C/A | 0.465 | 2.88E-05 | 29.7 | -0.037 | 0.007 | 4.79E-08 | 0.003 | 0.013 | 8.00E-01 |
| rs7096385 | 10:69664881 | SIRT1, MYPN | C/T | 0.908 | 2.87E-05 | 29.6 | -0.071 | 0.013 | 4.87E-08 | -0.017 | 0.018 | 3.71E-01 |
| rs7170477 | 15:64103777 | HERC1 | A/G | 0.304 | 2.89E-05 | 29.8 | 0.039 | 0.007 | 4.98E-08 | 0.001 | 0.013 | 9.46E-01 |

SNP, single nucleotide polymorphism; E/A, effect/alternative alleles; EAF, effect allele frequency; F, the F statistic for each SNP, was calculated as: F=R^2^×(n-2)/(1-R^2^); AF, atrial fibrillation; OSA, obstructive sleep apnea; SE, standard error.

**Table S3.** Characteristics of the SNPs significantly associated with potential confounders (P < 5×10^-8^).

| **Exposure** | **SNP** | **EA/NEA** | **Trait** | **Beta** | **P-value** |
| --- | --- | --- | --- | --- | --- |
| Obstructive sleep apnea | rs9937053 | A/G | Body mass index | 0.068 | 4.12E-174 |
|  |  |  | Type II diabetes | 0.129 | 3.50E-24 |
|  |  |  | Self-reported hypertension | 0.008 | 4.68E-13 |
| Atrial fibrillation | rs10006327 | C/T | Self-reported hypertension | -0.007 | 4.29E-10 |
|  | rs12245149 | A/C | Diastolic blood pressure | 0.018 | 1.04E-12 |
|  |  |  | Body mass index | 0.015 | 5.94E-10 |
|  | rs12604076 | C/T | Systolic blood pressure | -0.015 | 8.93E-10 |
|  |  |  | Weight | -0.014 | 1.02E-10 |
|  | rs1458038 | T/C | Self-reported hypertension | 0.020 | 1.08E-67 |
|  |  |  | Systolic blood pressure | 0.036 | 2.62E-41 |
|  |  |  | Diastolic blood pressure | 0.033 | 2.33E-34 |
|  | rs1563304 | C/T | Snoring | 0.009 | 4.64E-08 |
|  | rs2540949 | T/A | Systolic blood pressure | -0.018 | 4.67E-12 |
|  | rs284277 | C/A | Self-reported hypertension | 0.011 | 5.50E-24 |
|  |  |  | Systolic blood pressure | 0.018 | 6.77E-12 |
|  | rs2885697 | G/T | Weight | 0.017 | 8.45E-15 |
|  | rs35005436 | C/T | Self-reported hypertension | 0.008 | 1.38E-08 |
|  | rs422068 | C/T | Systolic blood pressure | -0.016 | 1.35E-10 |
|  | rs4951258 | A/G | Weight | 0.013 | 6.49E-10 |
|  | rs56201652 | A/G | Weight | 0.028 | 1.69E-30 |
|  |  |  | Systolic blood pressure | -0.016 | 3.59E-09 |
|  | rs60212594 | C/G | Systolic blood pressure | 0.023 | 3.26E-11 |
|  | rs9899183 | C/T | Systolic blood pressure | 0.015 | 2.22E-08 |
|  |  |  | Self-reported hypertension | 0.007 | 2.71E-08 |
| Snoring | rs2307111 | T/C | Body mass index | 0.029 | 6.35E-32 |
|  |  |  | Total cholesterol | -0.042 | 3.52E-30 |
|  |  |  | Low density lipoprotein | -0.042 | 7.34E-28 |
|  |  |  | Obesity | NA | 3.00E-12 |
|  | rs34811474 | A/G | Body mass index | -0.031 | 5.30E-27 |
|  |  |  | Diastolic blood pressure | -0.016 | 2.59E-08 |
|  | rs8069947 | C/T | Systolic blood pressure | 0.017 | 6.73E-12 |
|  | rs2049045 | C/G | Body mass index | -0.040 | 2.70E-38 |
|  |  |  | Weight | -0.031 | 2.40E-30 |
|  |  |  | Overweight | -0.081 | 2.30E-12 |
|  | rs6054427 | A/G | Body mass index | 0.020 | 5.90E-16 |

SNP, single nucleotide polymorphism; EA/NEA, effect allele/ none-effect allele.

The results were obtained from PhenoScanner v2 on July 5, 2021.

**Table S4.** Characteristics of the genetic variants used for Mendelian randomization analysis of snoring on atrial fibrillation.

| **SNP** | **Chromosome position** | **Nearest Gene** | **Alleles (E/A)** | **EAF** | **R^2^** | **F** | **SNP-snoring Associations** | | | **SNP-AF Associations (Nielsen)** | | |
| --- | --- | --- | --- | --- | --- | --- | --- | --- | --- | --- | --- | --- |
|  |  |  |  |  |  |  | **Beta** | **SE** | **P value** | **Beta** | **SE** | **P value** |
| rs592333 | 13:51340315 | DLEU7 | A/G | 0.442 | 1.82E-04 | 74.2 | -0.009 | 0.001 | 1.00E-17 | -0.001 | 0.007 | 8.49E-01 |
| rs10878269 | 12:65791463 | MSRB3 | C/T | 0.350 | 1.63E-04 | 66.5 | -0.009 | 0.001 | 2.30E-16 | 0.014 | 0.007 | 3.80E-02 |
| rs61597598 | 2:156996626 | AC073551.1 | G/A | 0.116 | 1.48E-04 | 60.5 | -0.012 | 0.002 | 5.10E-15 | -0.016 | 0.010 | 1.15E-01 |
| rs2307111 | 5:75003678 | POC5 | T/C | 0.396 | 1.26E-04 | 51.4 | 0.008 | 0.001 | 4.80E-13 | 0.013 | 0.007 | 5.92E-02 |
| rs2664299 | 14:99742187 | BCL11B | T/C | 0.415 | 1.23E-04 | 50.0 | 0.008 | 0.001 | 1.10E-12 | -0.001 | 0.007 | 8.77E-01 |
| rs13251292 | 8:71474355 | TRAM1 | A/G | 0.415 | 1.17E-04 | 47.7 | -0.007 | 0.001 | 4.30E-12 | 0.000 | 0.007 | 9.78E-01 |
| rs57222984 | 17:43758898 | CRHR1:RP11-105N13.4 | A/G | 0.265 | 1.17E-04 | 47.8 | -0.008 | 0.001 | 5.40E-12 | -0.040 | 0.008 | 1.20E-06 |
| rs725861 | 10:9063776 | RP11-42L9.2 | A/G | 0.192 | 1.13E-04 | 46.1 | -0.009 | 0.001 | 1.00E-11 | 0.002 | 0.009 | 8.51E-01 |
| rs12119849 | 1:96878072 | UBE2WP1 | G/A | 0.083 | 1.06E-04 | 43.4 | -0.012 | 0.002 | 4.10E-11 | -0.012 | 0.012 | 3.27E-01 |
| rs12429765 | 13:40745860 | LINC00332 | A/G | 0.493 | 1.03E-04 | 41.9 | 0.007 | 0.001 | 6.20E-11 | 0.007 | 0.006 | 2.83E-01 |
| rs34811474 | 4:25408838 | ANAPC4 | G/A | 0.217 | 1.02E-04 | 41.8 | 0.008 | 0.001 | 1.30E-10 | 0.025 | 0.008 | 2.92E-03 |
| rs7829639 | 8:78215352 | AC105242.1 | A/G | 0.297 | 1.01E-04 | 41.2 | -0.007 | 0.001 | 1.40E-10 | 0.041 | 0.019 | 2.85E-02 |
| rs180107 | 17:67930772 | AC002539.2 | A/T | 0.370 | 1.01E-04 | 41.1 | -0.007 | 0.001 | 2.10E-10 | -0.010 | 0.007 | 1.31E-01 |
| rs8069947 | 17:1985843 | SMG6 | C/T | 0.488 | 9.67E-05 | 39.5 | 0.007 | 0.001 | 2.80E-10 | 0.006 | 0.007 | 3.63E-01 |
| rs11018488 | 11:88861590 | AP001482.1 | A/T | 0.364 | 9.33E-05 | 38.1 | 0.007 | 0.001 | 5.30E-10 | 0.010 | 0.007 | 1.74E-01 |
| rs8108822 | 19:32183171 | RNA5SP471 | C/T | 0.102 | 9.11E-05 | 37.2 | 0.011 | 0.002 | 6.20E-10 | 0.001 | 0.011 | 9.63E-01 |
| rs4976269 | 5:134452597 | C5orf66 | G/A | 0.341 | 9.24E-05 | 37.7 | 0.007 | 0.001 | 8.60E-10 | 0.001 | 0.007 | 8.79E-01 |
| rs2049045 | 11:27694241 | BDNF-AS: BDNF | G/C | 0.191 | 9.47E-05 | 38.7 | 0.008 | 0.001 | 8.80E-10 | 0.009 | 0.009 | 3.27E-01 |
| rs9900496 | 17:7439739 | Y_RNA | T/C | 0.317 | 8.76E-05 | 35.8 | -0.007 | 0.001 | 1.80E-09 | -0.017 | 0.007 | 1.90E-02 |
| rs17151229 | 7:127382155 | SND1 | G/C | 0.354 | 8.62E-05 | 35.2 | -0.007 | 0.001 | 1.90E-09 | 0.014 | 0.007 | 4.14E-02 |
| rs6054427 | 20:6635266 | RP5-859D4.3 | G/A | 0.378 | 8.38E-05 | 34.2 | -0.006 | 0.001 | 4.00E-09 | -0.011 | 0.007 | 1.18E-01 |
| rs145367119 | 3:90049321 | U3 | C/T | 0.431 | 7.77E-05 | 31.7 | 0.006 | 0.001 | 1.20E-08 | 0.001 | 0.009 | 8.86E-01 |
| rs17060460 | 6:100827834 | SIM1 | A/G | 0.235 | 7.92E-05 | 32.3 | -0.007 | 0.001 | 1.40E-08 | -0.002 | 0.008 | 8.23E-01 |
| rs227727 | 17:54776955 | C17orf67 | A/T | 0.450 | 7.93E-05 | 32.4 | -0.006 | 0.001 | 1.40E-08 | -0.010 | 0.007 | 1.30E-01 |
| rs947612 | 6:73738661 | KCNQ5 | G/A | 0.229 | 7.57E-05 | 30.9 | 0.007 | 0.001 | 1.50E-08 | -0.002 | 0.008 | 7.92E-01 |
| rs80093081^†^ | 1:39820926 | MACF1 | T/G | 0.292 | 7.67E-05 | 31.3 | 0.006 | 0.001 | 1.80E-08 | -0.003 | 0.007 | 6.98E-01 |
| rs2207944 | 6:84307328 | SNAP91 | T/C | 0.457 | 7.64E-05 | 31.2 | -0.006 | 0.001 | 2.00E-08 | -0.008 | 0.007 | 2.54E-01 |
| rs6099273 | 20:55347828 | RNU6-929P | C/T | 0.235 | 7.52E-05 | 30.7 | -0.007 | 0.001 | 2.60E-08 | -0.003 | 0.008 | 7.35E-01 |
| rs4987719 | 18:60960310 | BCL2 | C/T | 0.028 | 7.63E-05 | 31.2 | -0.016 | 0.003 | 3.50E-08 | 0.011 | 0.024 | 6.49E-01 |
| rs4744369^*^ | 9:97475396 | C9orf3 | T/A | 0.396 | 7.29E-05 | 29.7 | -0.006 | 0.001 | 4.00E-08 | 0.074 | 0.007 | **1.83E-27** |
| rs9583546 | 13:111566412 | ANKRD10 | G/C | 0.369 | 7.22E-05 | 29.5 | -0.006 | 0.001 | 4.00E-08 | 0.007 | 0.007 | 3.49E-01 |

SNP, single nucleotide polymorphism; E/A, effect/alternative alleles; EAF, effect allele frequency; F, the F statistic for each SNP, was calculated as: F=R^2^×(n-2)/(1-R^2^); AF, atrial fibrillation; SE, standard error.

^*^ The SNP was not included in MR analyses due to genome-wide significant association with AF.

^†^ Proxy SNP rs597311 (r^2^=0.88) was identified for rs80093081.

**Table S5.** Results of causal inference between obstructive sleep apnea and atrial fibrillation.

| Exposures | | OSA | OSA | AF (Nielsen) | Snoring |
| --- | --- | --- | --- | --- | --- |
| Outcomes | | AF (Nielsen) | AF (AF HRC) | OSA | AF (Nielsen) |
| Number of SNPs | | 5 | 5 | 111 | 30 |
| IVW (fe) | OR (95% CI) | 1.210 (1.119-1.307) | 1.166 (1.074-1.267) | 1.022 (0.981-1.066) | 1.883 (1.305-2.717) |
|  | P-value | 1.51×10^-6^ | 2.63×10^-4^ | 0.294 | 7.11×10^-4^ |
|  | P_Cochran’s Q_ | 0.245 | 0.134 | 0.019 | 3.82×10^-4^ |
| IVW (mre) | OR (95% CI) | NA | NA | 1.022 (0.986-1.060) | 1.883 (1.104-3.214) |
|  | P-value | NA | NA | 0.232 | 0.02 |
| Weighted median | OR (95% CI) | 1.222 (1.100-1.358) | 1.217 (1.090-1.359) | 1.052 (0.981-1.127) | 1.366 (0.789-2.364) |
|  | P-value | 1.96×10^-04^ | 4.69×10^-4^ | 0.156 | 0.265 |
| MR-Egger | OR (95% CI) | 1.368 (1.122-1.668) | 1.146 (0.827-1.587) | 1.053 (0.972-1.140) | 1.731 (0.108-27.811) |
|  | P-value | 1.91×10^-03^ | 0.474 | 0.207 | 0.701 |
|  | P_intercept_ | 0.180 | 0.908 | 0.397 | 0.952 |
| MR-RAPS | OR (95% CI) | 1.214 (1.107-1.331) | 1.177 (1.071-1.295) | 1.027 (0.984-1.071) | 1.844 (1.143-2.974) |
|  | P-value | 3.64×10^-05^ | 7.57×10^-4^ | 0.224 | 0.012 |
| MR-PRESSO | OR (95% CI) | NA | NA | NA | 1.841 (1.184-2.863) |
|  | P-value | NA | NA | NA | 0.012 |
|  | P_MR-PRESSO global test_ | 0.240 | 0.192 | 0.023 | 4.00×10^-4^ |
|  | Number of outliers | 0 | 0 | 0 | 2 |
| IVW (fe)-Pleiotropic SNPs excluded | OR (95% CI) | 1.154 (1.047-1.271) | 1.128 (1.018-1.251) | 1.015 (0.977-1.054) | 1.519 (1.016-2.271) |
|  | P-value | 3.93×10^-03^ | 2.17×10^-2^ | 0.442 | 0.042 |
|  | Number of pleiotropic SNPs | 1 | 1 | 14 | 5 |

OSA, obstructive sleep apnea; AF, atrial fibrillation; SNPs, SNP, single nucleotide polymorphisms; OR, odds ratio; CI, confidence interval; IVW (fe), fixed-effects inverse-variance weighted; IVW (mre), multiplicative random-effects inverse-variance weighted; MR-RAPS, MR-robust adjusted profile score; MR-PRESSO, MR-pleiotropy residual sum and outlier; NA, not available.

# ****Supplementary Figures****

**Figure S1.** Leave-one-out analysis for causal effect of obstructive sleep apnea on atrial fibrillation.

**
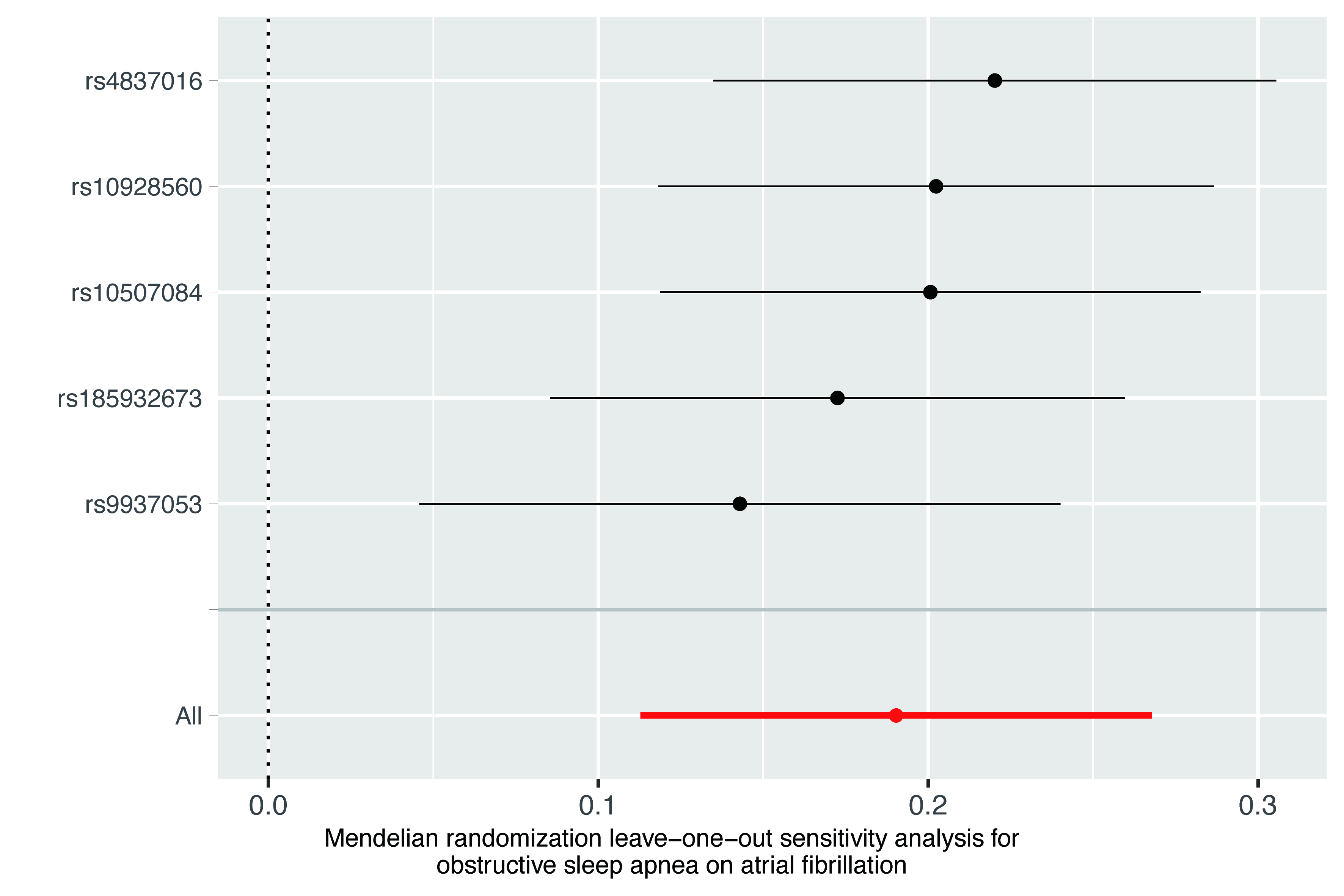
**

**Figure S2.** Additional Mendelian randomization analyses of the associations of snoring with atrial fibrillation.


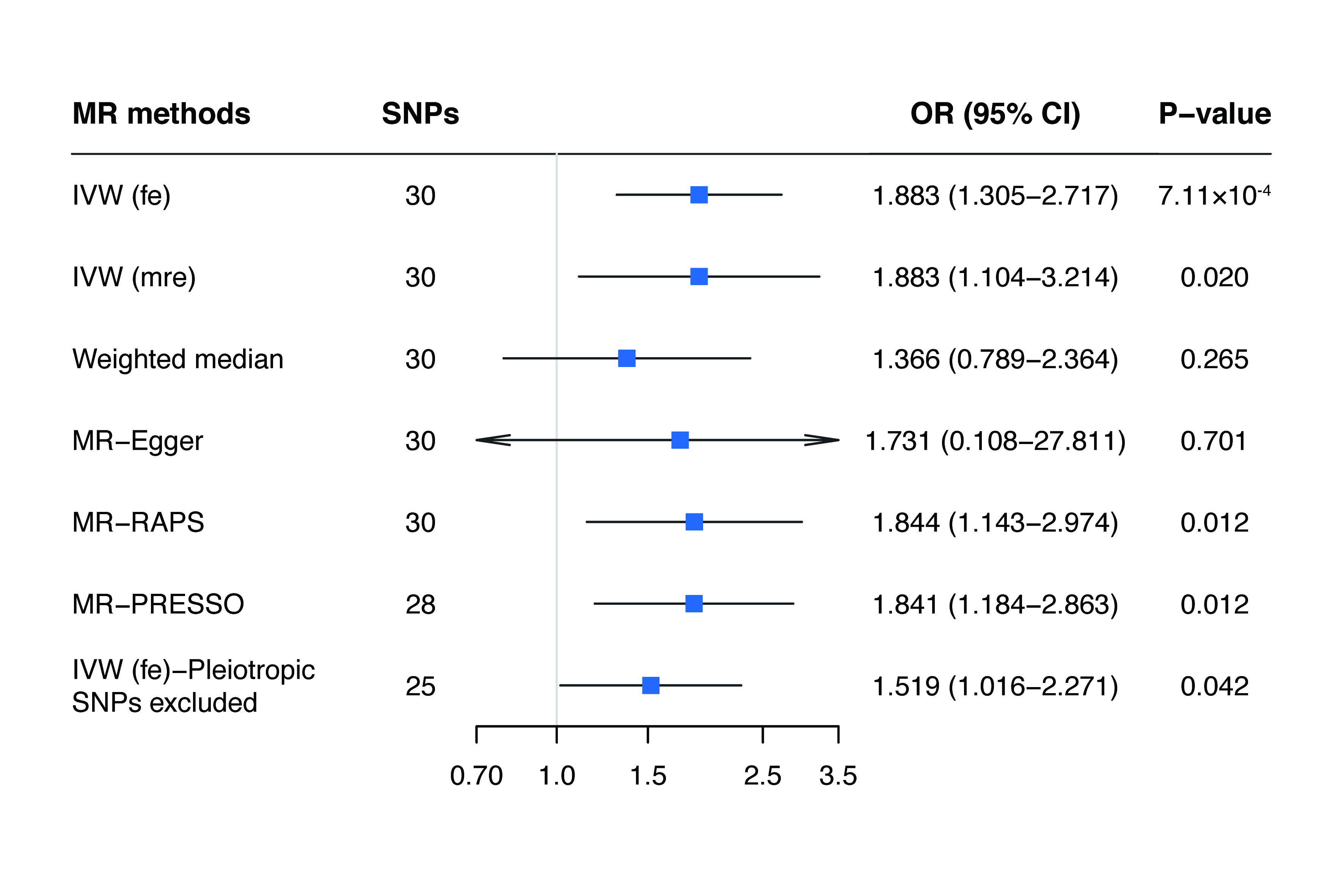


MR, Mendelian randomization; SNPs, single nucleotide polymorphisms; OR, odds ratio; CI, confidence interval; IVW (fe), fixed-effects inverse-variance-weighted; IVW (mre), multiplicative random-effects inverse-variance-weighted; MR-RAPS, MR-robust adjusted profile score; MR-PRESSO, MR-pleiotropy residual sum and outlier.

**Figure S3.** Leave-one-out analysis for causal effect of atrial fibrillation on obstructive sleep apnea.

^
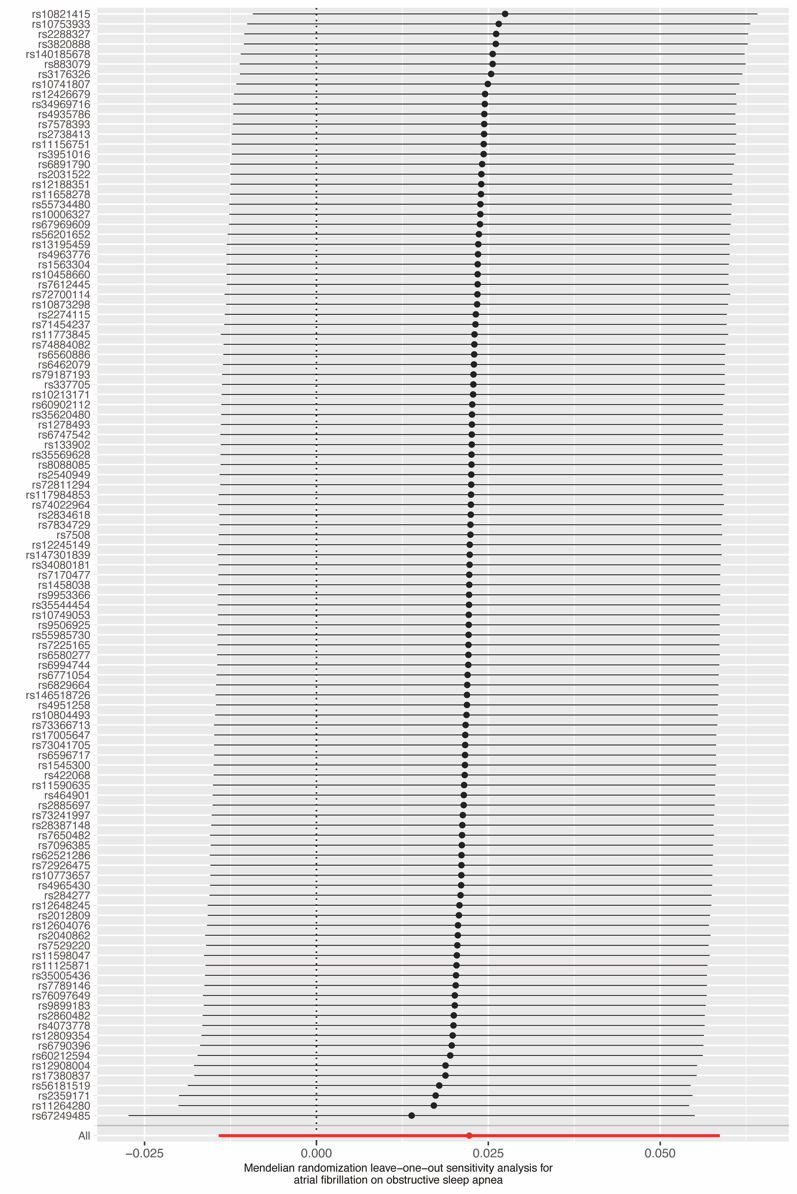
^
